# Physical Health Constraints and Psychological Distress in Older Asian Americans: The Role of Perceived Health

**DOI:** 10.1093/geroni/igaa057.1080

**Published:** 2020-12-16

**Authors:** Hyunwoo Yoon, Yuri Jang, Seoyoun Kim

**Affiliations:** 1 Portland State University, Portland, Oregon, United States; 2 University of Southern California, Los Angeles, California, United States; 3 Texas State University, San Marcos, Texas, United States

## Abstract

Given the importance of understanding the underlying dynamics of physical and mental health in old age, the present study explored the roles of physical health constraints in predicting subjective health perception and psychological distress among older Asian Americans. Guided by the Health Belief Model, we also examined whether subjective health perception would function as a mediator in the link between physical health constraints and psychological distress. Using data from 533 Asian Americans aged 60 and over (mean age=69.4, SD=6.88) in the 2016 Asian American Quality of Life Study, the direct and indirect effect models were tested with multivariate linear regressions and the PROCESS macro. Advanced age, unmarried status, lower levels of acculturation, and more chronic physical conditions were significant predictors of psychological distress. When subjective health perception was added to the model, an additional 5% of the variance was accounted for, resulting in 25% of the total variance explained by the estimated model. Negative health perception was a significant predictor of increased level of psychological distress. Supporting the mediation hypothesis, all direct paths among physical health constraints, subjective health perception, and psychological distress were significant. The indirect effect of physical health constraints on psychological distress through subjective health perception status was significant, as evidenced by the 95% bootstrap confidence interval for the indirect effect not containing zero (.07, .28). The findings not only help better understand the psychological mechanisms that underlie physical health constraints and psychological distress but also suggest avenues for interventions.

